# Experimenting with reproducibility: a case study of robustness in bioinformatics

**DOI:** 10.1093/gigascience/giy077

**Published:** 2018-06-28

**Authors:** Yang-Min Kim, Jean-Baptiste Poline, Guillaume Dumas

**Affiliations:** 1 Human Genetics and Cognitive Functions Unit, Institut Pasteur, 25 rue du Docteur Roux 75015 Paris, France; 2 CNRS UMR 3571 Genes, Synapses and Cognition, Institut Pasteur, 25 rue du Docteur Roux 75015 Paris, France; 3 Paris Diderot University, Sorbonne Paris Cité, 5 rue Thomas Mann 75013 Paris, France; 4 Center of Bioinformatics, Biostatistics and Integrative Biology (C3BI), USR 3756, Institut Pasteur and CNRS, 25-28 rue du Docteur Roux 75015 Paris, France; 5 Montreal Neurological Institute and Hospital, Brain Imaging Center, Ludmer Center, McGill University, 3801 University Street, Montreal, QC H3A 2B4, Canada; 6 Henry H. Wheeler, Jr. Brain Imaging Center, Helen Wills Neuroscience Institute, 132 Barker Hall, office 210S, MC 3190, University of California, Berkeley, CA 94720, USA

**Keywords:** reproducibility, robustness, reusability, network based stratification, standard consensus dataset, cancer

## Abstract

Reproducibility has been shown to be limited in many scientific fields. This question is a fundamental tenet of scientific activity, but the related issues of reusability of scientific data are poorly documented. Here, we present a case study of our difficulties in reproducing a published bioinformatics method even though code and data were available. First, we tried to re-run the analysis with the code and data provided by the authors. Second, we reimplemented the whole method in a Python package to avoid dependency on a MATLAB license and ease the execution of the code on a high-performance computing cluster. Third, we assessed reusability of our reimplementation and the quality of our documentation, testing how easy it would be to start from our implementation to reproduce the results. In a second section, we propose solutions from this case study and other observations to improve reproducibility and research efficiency at the individual and collective levels.

While finalizing our code, we created case-specific documentation and tutorials for the associated Python package *StratiPy*. Readers are invited to experiment with our reproducibility case study by generating the two confusion matrices (see more in section “Robustness: from MATLAB to Python, language and organization"). Here, we propose two options: a step-by-step process to follow in a Jupyter/IPython notebook or a Docker container ready to be built and run.

## Background

The collective endeavor of science depends on researchers being able to replicate the work of others. In a recent survey of 1,576 researchers, 70% of them admitted having difficulty in reproducing experiments proposed by other scientists [1]. For 50%, this reproducibility issue even concerns their own experiments. Despite the growing attention on the replication crisis in science [2, 3], this controversial subject is far from being new. Even in the 17th century, scientists criticized the air pump invented by physicist Robert Boyle because it was too complicated and expensive to build [4].

**Figure 1:**
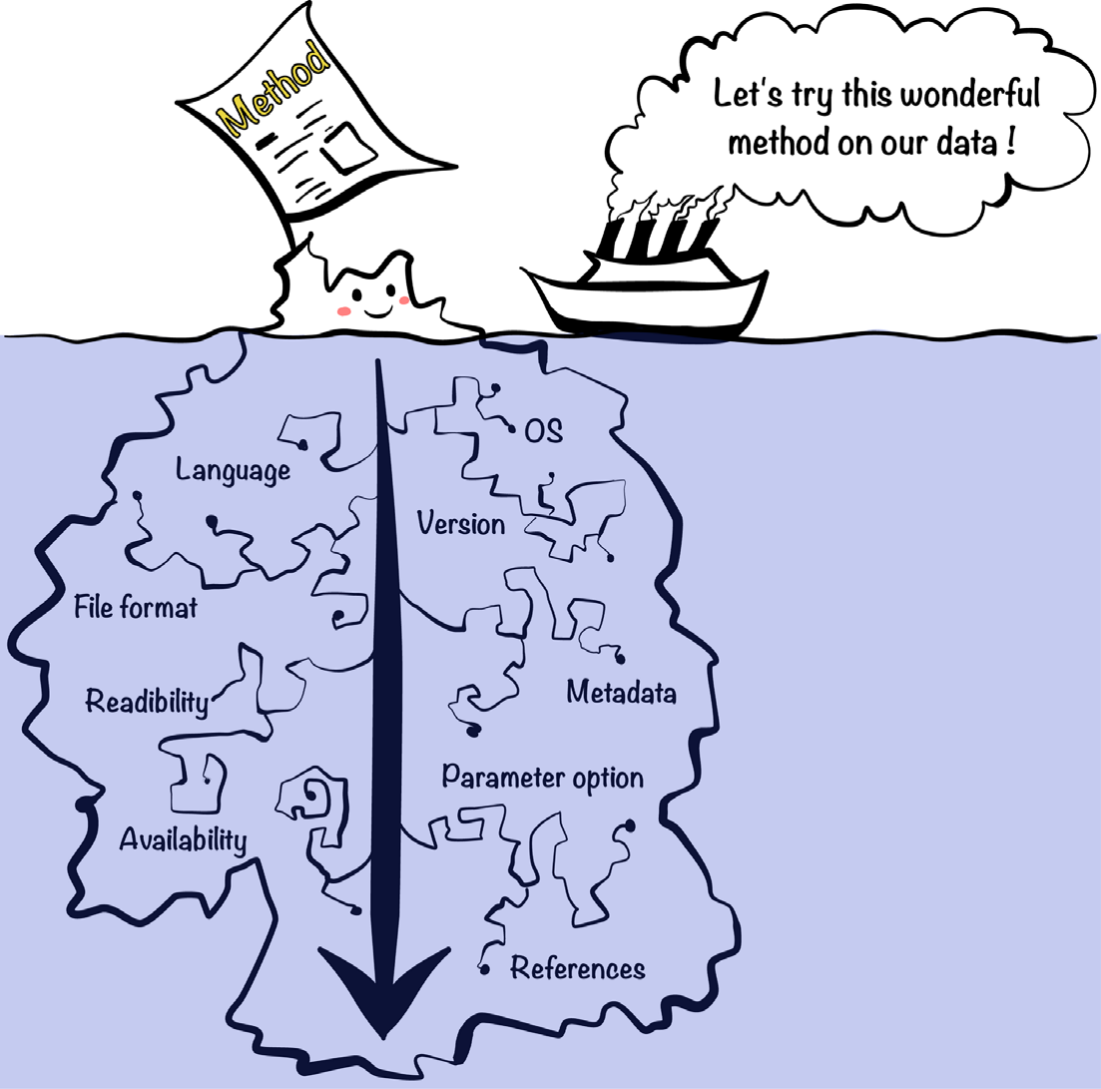
Hidden reproducibility issues are like an underwater iceberg. Scientific journal readers have the impression that they can almost see the full work involved in the method. In reality, articles do not take into account adjustment and configuration for significant replication in most cases. Therefore, there is a significant gap between apparent executable work (i.e., above water portion of iceberg) and necessary effort in practice (i.e., the full iceberg).

Several concepts for reproducibility in computational science are closely associated [5, 6]. Here, we define them as mentioned by Whitaker [6]. First, obtaining the same results using the same data and same code is “reproducibility". If code is different, it is “robustness". If we used different data but with the same code, it is “replicability". Last, using different data and different code is referred as “generalizability". Here, we primarily elaborate on reproducibility and robustness and acknowledge that new datasets or hardware environments introduce additional hurdles [7]. Reproducibility is a key first step. For example, among the 400 algorithms published during the major artificial intelligence conferences, only 6% offered the code [8]. Even when authors provide data and code, the outcome can vary either marginally or fundamentally [9]. Tackling irreproducibility in bioinformatics thus requires considerable effort beyond code and data availability, an effort that is still poorly recognized in the current publication-based research community. In most cases, there is a significant gap between apparent executable work (Fig. 1 - i.e., the above-water portion of iceberg) and necessary effort in practice (Fig. 1 - i.e., the full iceberg). Such effort is necessary to increase the consistency of the literature and the efficiency of the scientific research process by making research products easily reusable.

## Reproducibility and robustness in bioinformatics: a case study

### Reproducibility: from MATLAB to MATLAB, OS and environment

Our team studies autism spectrum disorders, a group of neurodevelopmental disorders well known for their heterogeneity. One of the current challenges of our research is to uncover homogeneous subgroups of patients (i.e., stratification) with more precise clinical outcomes, improving their prognosis and treatment [10, 11]. An interesting stratification method was proposed in the field of cancer research [12], where the authors proposed combining genetic profiles of patients’ tumors with protein-protein interaction networks in order to uncover meaningful homogeneous subgroups, a method called network-based stratification (NBS).

Before using the NBS method on our data, we studied the method by reproducing results from the original study. We are very grateful to the main authors who kindly provided all the related data and code online and gave us invaluable input upon request. The authors of this study did much more to help reproduce their results than is generally done. Despite their help, we experienced a number of difficulties that we document here, hoping that this report will help future researchers improve the reproducibility of results and reusability of research products.

Our first step was to execute the original method code with the given data: reproducibility (Table 1). To improve execution speed, the original authors used a library for MATLAB on a Linux platform, using executable compiled code MEX file [13], i.e., MTIMESX [14], a library allowing acceleration of large matrix multiplication. MEX files, however, are specific to the architecture and have to be recompiled for each operating system (OS). Since our lab was using Mac OS X Sierra, the compilation of this MEX file into a mac64 binary required a new version of MTIMESX. It was also necessary to install and configure properly OpenMP [15], a development library for parallel computing. After this, the original MATLAB code was successfully run in our environment.

**Table 1:** Technical problems encountered during our reproducibility and robustness case study

	Code	Data	Technical issues	Other issues
Reproducibility	Same: MATLAB	Same	**OS**: MEX file specificity linked to OS (e.g., Linux → OSX)	
Robustness	MATLAB to Python	Same	**File format**: we can load sparse matrices from *.mat* file but cannot save them into HDF5 using h5py package**Default parameters**: e.g linkage methods of hierarchical clustering with• MATLAB (MathWorks) using Unweighted Pair Group Method with Arithmetic Mean (UPGMA) and • Python (SciPy) using single method	• Metadata structure• Important parameter value not explained in the original paper• Remaining discarded work (“code ruins”) and traces of debugging
Reproducibility of Robustness	Same: Python	Same	**OS:** Numpy package and basic linear algebra subprogram library compiled for specific OS (e.g., OSX → Linux)	Documentation

These issues are classic but may not be overcome by researchers with little experience in compilation or installation issues. For these reasons alone, many individuals may turn down the opportunity of reusing code and therefore the method.

In the next section, we focus on code re-implementation, a procedure that can increase understanding of the method but is even more time consuming.

### Robustness: from MATLAB to Python, language and organization

To fully master the method, we developed a complete open-source toolkit of genomic stratification in Python [16]. Python is also an interpreted programming language. However, contrary to MATLAB, it is free and has a GNU General Public License (GPL) -compatible license [17], which fosters both robustness and generalizability. Recoding in another language in a different environment will lead to some unavoidable problems such as variation in low-level libraries (e.g., glibc); it is likely that the outcomes will vary even if the same algorithm is implemented [18]. In addition, we rely on Python packages to perform visualization or linear algebra computations (e.g., Matplotlib, SciPy, NumPy [19–21]), and results may depend on these packages' versions.  Python is currently in a transitional period between two major versions, 2 and 3. We chose to write the code in Python 3, which is the current recommendation.

#### Metadata and file formats

Even if the original code could be run, we had to handle several file formats to check and understand the structure of the original data. For instance, the data were provided by the Cancer Genome Atlas (TCGA) [22] and made available in a MATLAB *.mat* file format v7.2 as compressed data (sparse matrices). Thanks to SciPy, Python can load the MATLAB files version. We wanted to use the open format HDF5 to save the results; however, Scipy's sparse matrices could not be stored in HDF5 format (Table 1). We thus decided to continue saving in *.mat* format. Moreover, the original authors had denoted download dates of patients’ data of TCGA, thereby clarifying the data provenance. However, in the absence of structural metadata, we had to explore the hierarchical structure of the variables (e.g., patient ID, gene ID, phenotype).

#### Codes and parameters

**Figure 2:**
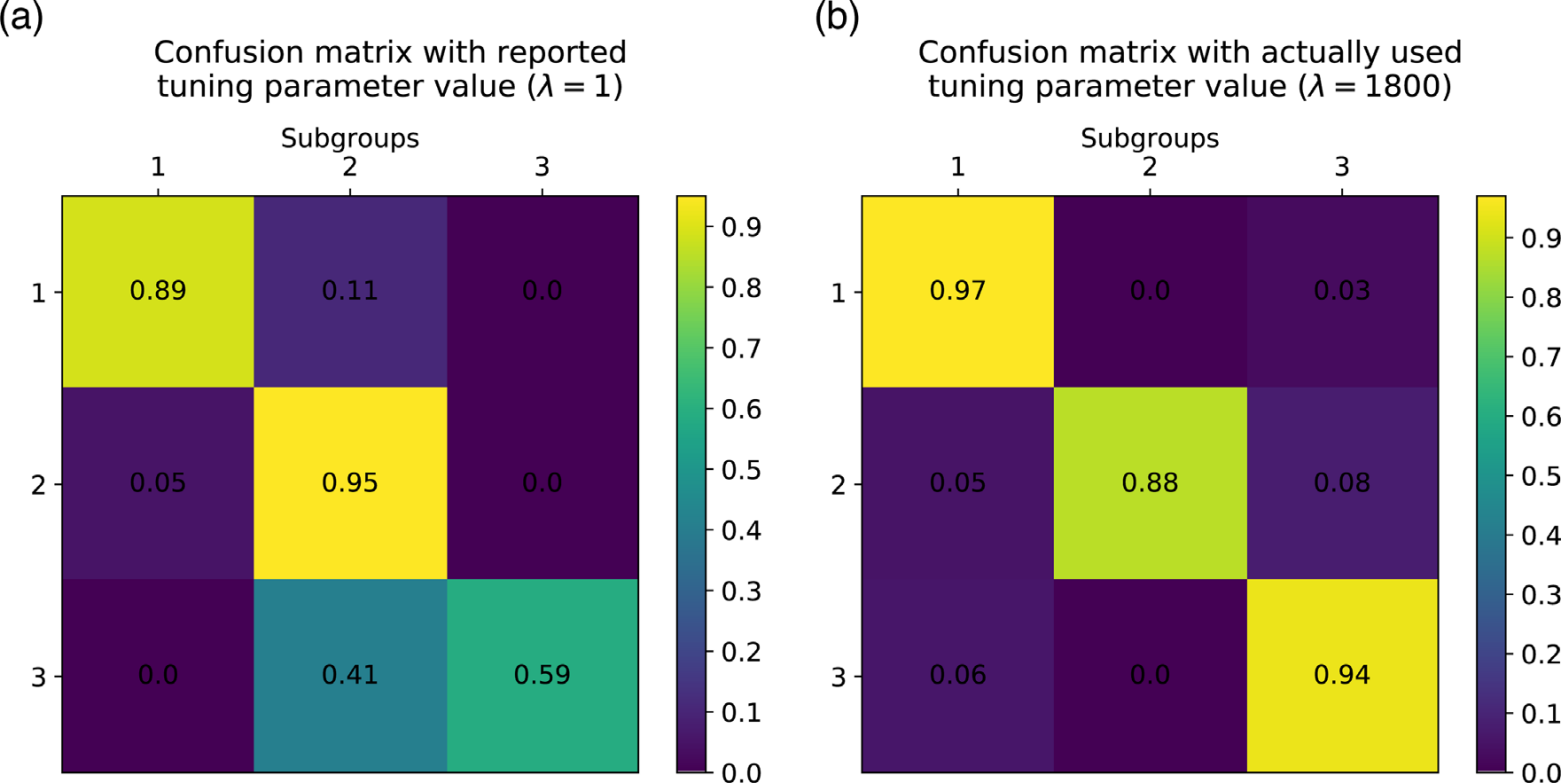
Normalized confusion matrices between original and replicated results. Before **(a)** and after **(b)** applying the appropriate value of the graph regularization factor on the NBS method. Each row or column corresponds to a subgroup of patients (here three subgroups). The diagonal elements show the frequency of correct classifications for each subgroup. A high value indicates a correct prediction.

Beyond documentation and file formats, code initialization and parameter settings are also keys for reproducibility. Upon execution of the code, “unexpected” results were obtained. One cause was application of the hierarchical clustering step for which we used the clustering tools of SciPy. Both SciPy and MATLAB (MathWorks) functions offer seven linkage methods. However, SciPy's default option (single method) [23] differs from MATLAB's default option (Unweighted Pair Group Method with Arithmetic Mean: UPGMA) [24], which was used in the original study (Table 1). Another cause for the variation in results is the value of one of the most important parameters of the method, the graph regulator factor, which was not clarified in the original article. From the article, we believed that this factor had a constant value of 1.0 until we found that in the original code its value varies across iterations and converges to an optimal value around 1,800. Therefore, we initially obtained very different results from the original NBS (Fig. 2 - a) with heterogeneous subgroups. Once the optimal value was set up, we finally observed homogenous clusters (Fig. 2 - b). Moreover, during our attempts to run the original code in order to understand the causes of the errors, we realized that some parts of the code were not run any longer (e.g., discarded work, remaining traces of debugging), which made understanding the implementation harder.

To allow others to reproduce our results, we wrote some documentation and tutorials for the Python package *StratiPy* [16]. Readers are invited to experiment with reproducibility by generating the two confusion matrices of Fig. 2. This is described by the following tools: GitHub, Docker, and Jupyter/IPython notebook.

#### Documentation and examples

During the recoding process, we used an enhanced Python interpreter, IPython, which is an interactive shell supporting both Python 2 and 3, to debug. Since the dataset is large and the execution takes a significant amount of time, we used IPython to re-run interactively some subsections of the script, which is one of the most helpful features. IPython can be integrated in the web interface Jupyter Notebook, offering an advanced structure for mixing code and documentation. While the Jupyter/IPython notebook was initially convenient, it does not scale well to large programs and is not well adapted to versioning. However, the ability to mix code with document text is very useful for tutorials. A user of the code can read documentation (docstring) and text explanations and see how to run the code, explore parameters, and visualize results in the browser. Our work on NBS, as related here, can be reproduced with a Jupyter/IPython notebook available via our GitHub repository [16]. One can find more examples and several helpful links via this “gallery of interesting Jupyter Notebooks” [25], which contains a section about “Reproducible academic publications.”

**Figure 3:**
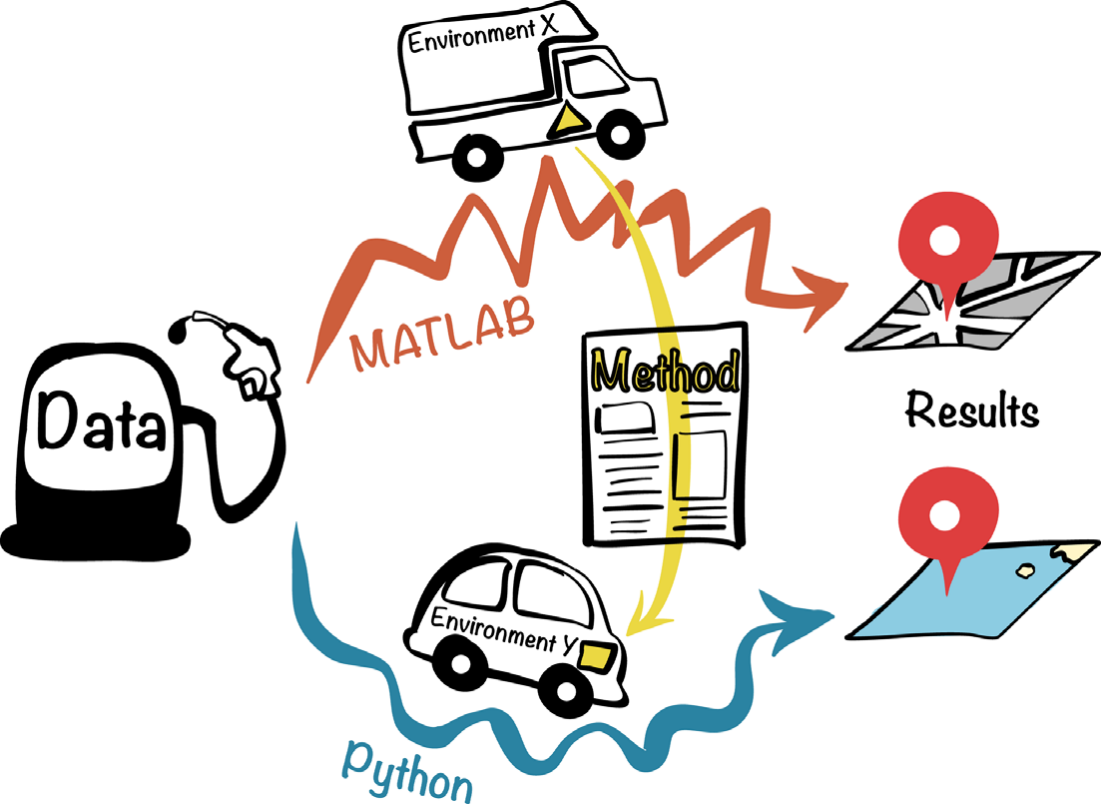
Analogy between robustness issues and road transport. The aim is to achieve the same output (i.e., to reach the same location) using published methods (i.e., engine). Despite the same input data (i.e., gasoline), we obtained different results due to different programming languages, e.g., MATLAB and Python (i.e., different roadways), and different environments (i.e., different vehicles).

To conclude, we were able to test the robustness of the method with our Python implementation. However, this took approximately two months for a senior researcher and six months for a master student. Fig. 3 illustrates this work through an analogy between robustness issues and road transport. Driving in a different environment (e.g., OS), we attempt to obtain identical results (i.e., to reach the same location) using the same input data (i.e., gasoline), but with a different computational environment (i.e., cars), different implementation of the method (i.e., engine), and different programming languages (i.e., MATLAB and Python roads).

### Collaborative code development and best practices

Throughout the project we used the version control system (VCS) Git to document the development of our Python package. Git is arguably one of the most powerful VCSs, allowing easy development of branches and allowing the distributed team (Paris, Berkeley, Montreal) to work collaboratively on the project. This project, *StratiPy*, is hosted on GitHub, a web-based Git repository hosting service [16]. While the original code was not available on GitHub, the main authors shared their code on a website. This should be sufficient for reproducibility and replicability for our purposes but makes it less easy to collaborate on the code. While working on our GitHub repository, researchers from the USA, India, China, and Europe contacted us about our robustness experiment. GitHub not only supports a better organization of projects, it also facilitates the collaboration on open-source software projects, thanks to its social network functions [26]. We adopted open-source coding standards and learned how to efficiently use Git and GitHub. Both required considerable training in the short-term, but brought clear benefits in the long-term, especially regarding collaboration and debugging.

### Reproducibility of robustness: from Python to Python

Knowing how difficult it can be to re-run someone else's code, we then attempted to start the analysis from scratch and to reproduce the results on another platform from our newly developed Python package. While the Python code was developed under Mac OS X Sierra (10.12), we used an Ubuntu 16.04.1 (Xenial) computer to test the Python implementation. Some additional issues emerged (Table 1). First, our initial documentation did not include the list of the required packages and instructions to launch the code. Second, the code was very slow to the extent that it was impractical to run it on a laptop because the Numpy package had not been compiled with a basic linear algebra subprogram that speeds up low-level routines that perform basic vector and matrix operations. Last, there was (initially) no easy way to check whether the results obtained on a different architecture were the expected ones. We added documentation and tests on the results files md5sum to solve this. To summarize, although the reuse and reproducibility of the results of the developed package were improved, these were far from being optimal in the first attempt.

## Potential Solutions: From Local to Global

### Act locally: simple practices and available tools

We conclude this reproducibility case study experiment by suggesting tools and best practices following the programming best practices of Wilson et al., such as modularizing and re-using code, unit testing, document design, data management, and project organization [2, 27], as well as keeping data provenance and recording all intermediate results [28].

#### Publish software and their environment

Increased reproducibility and replicability can be obtained by following Buckheit and Donoho's long-standing motto: “When we publish articles containing figures which were generated by computer, we also publish the complete software environment which generates the figures” by offering a complete and free package (WaveLab) to reproduce the published output [29]. Container and virtual machine technologies such as Docker [30], Vagrant [31], and Singularity [32, 33] (easily works in cluster environments) are becoming a standard solution to mitigate installation issues. These rely, however, on competencies that we think too few biologists possess today. While a container might encapsulate everything needed for a software execution, it can be hard to develop in a container. For instance, running Jupyter/IPython notebooks in Docker containers requires knowledge on advanced port forwarding, which may be discouraging for many biologists. Therefore, we propose two options in our example implementation of reproducibility: a step-by-step process to follow in a Jupyter/IPython notebook or a Docker container ready to be built and run. Mastering Docker, or other container tools, is increasingly becoming an important skill for biologists who use computational tools.

#### Document with appropriate metadata

Standard metadata are vital for efficient documentation of both data and software. In our example, we still lack the standard lexicon to document the data as well as document the software. However, we aim to follow the recommendations of Stodden et al. [34]: “Software metadata should include, at a minimum, the title, authors, version, language, license, uniform resource identifier/digital object identifier (DOI), software description (including purpose, inputs, outputs, dependencies), and execution requirements.” The more comprehensive the metadata description, the more likely the reuse will be both efficient and appropriate [35].

#### Write readable code

We draw the following conclusions from our experience in working with others code. First, the structure of the program should be clear and easily accessible. Second, good, concise code documentation and naming convention will help readability. Third, the code should not contain leftovers of previously tested solutions. When a solution takes a long time to compute, an option to store it locally can be proposed. Use of standard coding and documentation conventions (e.g., PEP 8 and PEP 257 in Python [36, 37]) with detailed comments and references of articles makes the code more accessible. When an algorithm is used, any modification from the original reference should be explained and discussed in the article as well as in the code. We advocate for researchers to write code “for their colleagues,” hence, ask for the opinion and review of co-working or partner laboratories. Furthermore, the collaboration between researchers working in different environments can more easily isolate reproducibility problems. In the future, journals may consider review of code as part of the standard review process [38].

#### Test the code

To check if the code is yielding a correct answer, software developers associate test suites (unit tests or integration tests) with their software. While we developed only a few tests in this project, we realized that this practice has a number of advantages, such as checking if the software installation seems correct and checking if updates in the code or in the operating system impact the results. In our case, we propose to check for the integrity of the data and for the results of some key processing.

### Think Globally: From Education to Community Standards

#### Training the new generation of scientists on digital tools and practices

The training for coding and software development is still too limited for biologists. Often, it is limited to self-training from searching answers on Stack Overflow or equivalent. Despite efforts by organizations such as Software [39] or Data Carpentry [40] and the growing demand for “data scientists” in the life sciences, university training and assessment on coding practices is still not generalized. The difficulty in accessing and understanding code may lead to applying code blindly without checking the validity of the results. Often, scientists prefer to believe that results are correct because checking the validity of the results may require significant time. Mastering a package such that results are truly understood can take a long time, as was the case in our experiment.

Academia could, and we argue should, instruct young scientists in best practices for reproducibility. For instance, Hothorn and Leisch organized a reproducibility workshop, gathering mostly PhD students and young post-docs specialized in bioinformatics and biostatistics. Then, they evaluated 100 random sample papers from *Bioinformatics* [3]. Their study revealed how such a workshop can raise young scientists' awareness about *“*what makes reproduction easy or hard at first hand.” Indeed, they found out that only a third of the original papers and two-thirds of applications notes had given access to the source code of the software used.

#### Standard consensus dataset and testing ecosystem

Here, we propose that publications related to bioinformatics methods are systematically accompanied with a test dataset, code source, and some basic tests (given ethical and legal constraints). As the method is tested on new datasets, the number of tests and range of applications would expand. We give a first example with our NBS re-implementation.

**Figure 4:**
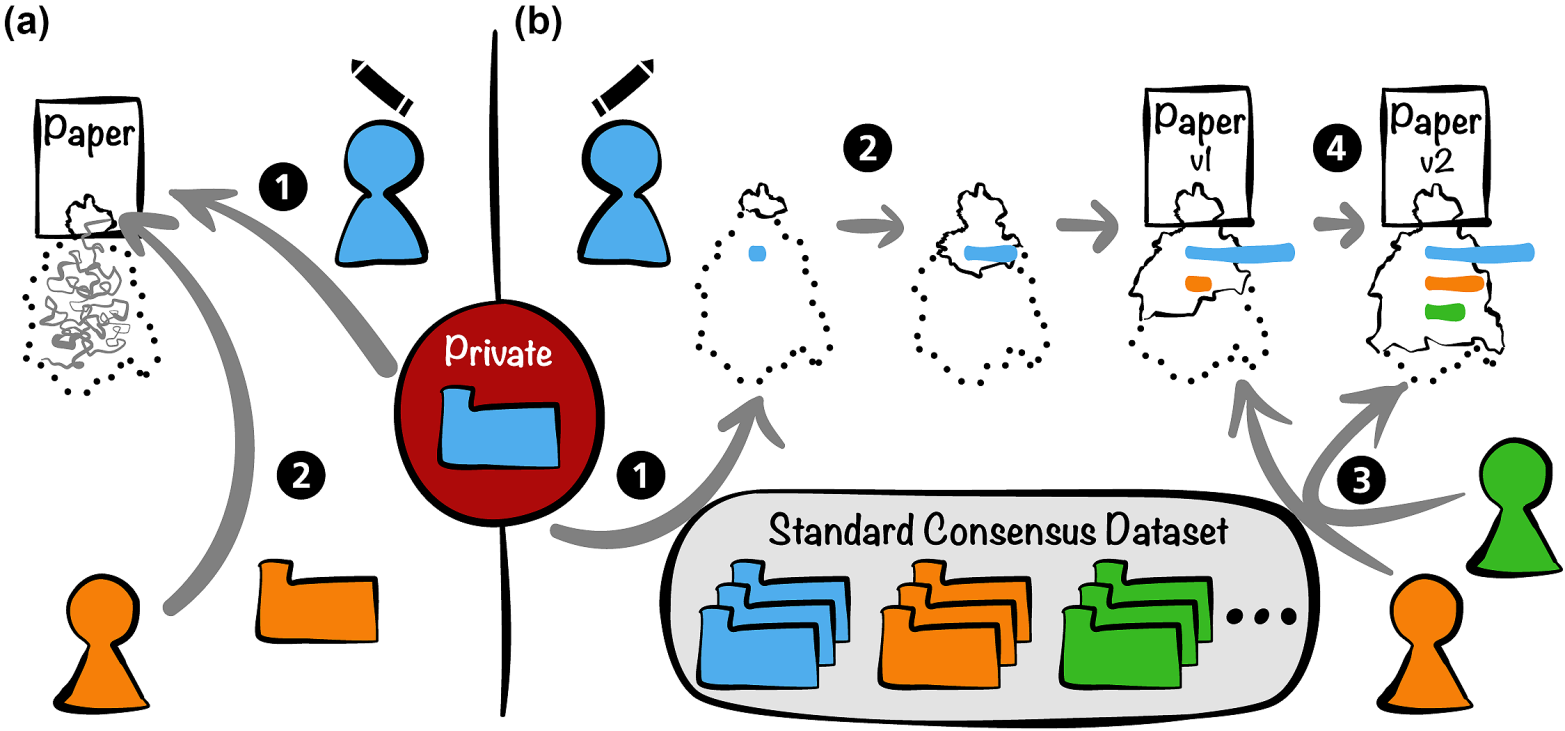
Working principles of testing ecosystem with private data. **(a)** A classic case: *(a.1)* Authors take private data (e.g., blue data) and then publish their method and corresponding results. *(a.2)* Users who have their own data (e.g., orange data) find a relevant paper but will be lost in the labyrinth of reproducibility. **(b)** Testing ecosystem with standard consensus dataset: *(b.1)* If authors work with their own data, they must identify corresponding standard data tag(s) (e.g., blue data). *(b.2)* Authors start to develop their method with corresponding standard data, and a reproducibility profile will be progressively built (bar length on iceberg corresponds to progression of replication test.) *(b.3)* Users can test a proposed method with other standard data (e.g., orange and green data) and thus participate to enhance the reproducibility profile. *(b.4)* Thanks to the collective work on testing, the method could be optimized and authors can upgrade their initial article (versioning).

A schematic overview of a possible testing ecosystem generalizing our test study is shown in Fig. 4. The core of this system would be a set of standard consensus datasets used to validate methods. For instance, in the field of machine learning, standard image databases are widely used for training and testing (e.g., MNIST for handwritten digits [41]). In the case of our proposal, data could be from different categories such as binary, text, image (shown as folders in different colors, Fig. 4 - b), and subcategories to introduce criteria such as size, quantitative/qualitative, and discrete/continuous, using a tagging system. Datasets could be issued from simulations or from acquisition and would validate a method on a particular component. This testing ecosystem would help scientists who cannot release their data because of privacy issues (Fig. 4 - a.1), although this can often be overcome, but also give access to data and tests to a wide community including establishments with limited financial resources.

We divide those who interact with scientific software or analysis code into two broad categories. First, the authors who propose a method and need to verify its validity and usefulness with open and/or private data. Second, the users (e.g., developers, engineers, bioinformaticians) who need to test and evaluate the proposed methods with other data.

When authors propose a new method, we propose that authors and users progressively build its reproducibility profile ( Fig. 4 - b.3, b.4) to document what method works best with what data. During the optimization of a project, the software code and associated documentation should be accessible to foster collaboration on additional use cases and data. When the work reaches some level of maturity, a complete article can be posted on a preprint server such as bioRxiv [42, 43] and be associated with a GitHub/GitLab repository with a DOI. With considerable effort, Stodden et al. conducted a reproducibility study on 204 random articles that appeared in *Science*. Despite some availability of the code, it had often been changed after publication, causing difficulties in replication [44]. In our proposed testing ecosystem, users will be able to launch reproducibility projects more easily thanks to code and article versioning.

Users who test and approve reproducibility on original or new data could be accredited and recognized by the scientific and developer communities (i.e., Stack Overflow, GitHub). This testing ecosystem could facilitate collaborations between methodology development and biological research communities.

## Conclusions and Perspective

In the 19th century, Pasteur introduced a detailed “Methods” section in his report. This advanced approach was necessary to reproduce his experiments and became the norm in the practice of science [45]. Today, with the advent of computational science, the reproducibility issue is seen as a growing concern. To summarize, our experiment at reproducing initial results led to the following recommendations:

- Improve life scientists software development skills.
- Use online repositories and tools to help other scientists in their exploration of the method [25, 26, 30].
- Enhance the cooperation between academia and industry [39, 40, 46].
- Develop an open-source continuous testing ecosystem with community standards, well-identified datasets to validate tools across versions and datasets, and go beyond the publication of a PDF file.

Verifying a previously published method can be very time consuming and is often poorly acknowledged. Some top-down initiatives already provide some incentives for such a process, e.g., the Horizon 2020 (H2020) [47] project of the European Commission (EC) that mandates open access of research data while respecting security and liability. H2020 supports OpenAIRE [48], a technical infrastructure of the open access, that allows the interconnection among projects, publications, datasets, and author information across Europe. Thanks to common guidelines, OpenAIRE interoperates with other web-based generalist scientific data repositories such as Zenodo, hosted by CERN, which allows the combination of data and GitHub repository via DOIs. The Open Science Framework also hosts data and software for a given project [49]. Respecting standard guidelines to transparently communicate the scientific work is a key step toward tackling irreproducibility and ensures a robust scientific endeavor.

## Key points

- The main barrier to reproducibility is the lack of compatibility among environments, programming languages, software versions, and the like.
- At the individual level, the key is research practices such as well-written, tested, and documented code; well-curated data; and the use of online repositories and collaborative tools.
- At the community level, we propose a testing ecosystem where standard consensus datasets are used to validate new methods and foster their generalizability.

## Availability of supporting data

The latest version of StratiPy (Python) with two examples of reproducibility and dataset are available at GitHub [https://github.com/GHFC/Stratipy; 16] and archived via a Zenodo DOI [50].

## Abbreviations

DOI: digital object identifier; H2020: Horizon 2020; NBS: network-based stratification; OS: operating system; TCGA: the Cancer Genome Atlas; VCS: version control system.

## Ethics approval and consent to participate

We used the uterine endometrial carcinoma dataset downloaded on 1 January 2013 from the TCGA portal as used by Hofree and colleagues in their previous paper [12].

## Competing interests

The authors declare that they have no competing interests.

## Funding

This work was supported by the following: Institut Pasteur (http://dx.doi.org/10.13039/501100003762); H2020 Societal Challenges (http://dx.doi.org/10.13039/100010676); Centre National de la Recherche Scientifique (http://dx.doi.org/10.13039/501100004794); Université Paris Diderot (http://dx.doi.org/10.13039/501100005736); Conny-Maeva Charitable Foundation; Cognacq-Jay Foundation; Orange (http://dx.doi.org/10.13039/501100003951); Fondation pour la Recherche Médicale (http://dx.doi.org/10.13039/501100002915); GenMed Labex; and BioPsy Labex. J.-B.P. was partially funded by NIH-NIBIB P41 EB019936 (ReproNim) NIH-NIMH R01 MH083320 (CANDIShare) and NIH 5U24 DA039832 (NIF), as well as the Canada First Research Excellence Fund, awarded to McGill University for the Healthy Brains for Healthy Lives initiative.

## Author contributions

Y-M.K., J-B.P., and G.D. wrote the manuscript. Y-M.K. and G.D. developed the StratiPy module. Y-M.K. was responsible for conceptualization, software, validation, writing the original draft, and reviewing and editing the manuscript. J.B.P. was responsible for validation, writing the original draft, and reviewing and editing the manuscript. G.D. was responsible for conceptualization, software, supervision, validation, writing the original original draft, and reviewing and editing the manuscript. All authors read and approved the final manuscript.

## Supplementary Material

GIGA-D-17-00317_Original_Submission.pdf

GIGA-D-17-00317_Revision_1.pdf

GIGA-D-17-00317_Revision_2.pdf

Response_to_Reviewer_Comments_Original_Submission.pdf

Response_to_Reviewer_Comments_Revision_1.pdf

Reviewer_1_Report_(Revision_1) -- Yi Zhang 4/28/2018 Reviewed

Reviewer_1_Report_(Original_Submission) -- Yi Zhang 1/31/2018 Reviewed

Reviewer_2_Report_(Revision_1) -- Franco Pestilli 4/23/2018 Reviewed

Reviewer_2_Report_(Original_Submission) -- Franco Pestilli 2/25/2018 Reviewed
