# Supplementary material for: Experimenting with reproducibility: a case study of robustness in bioinformatics
Source: Gigascience. 2018 Jun 28;7(7):giy077. doi: 10.1093/gigascience/giy077 (PMC6054242; doi:10.1093/gigascience/giy077)
Supplement: Response_to_Reviewer_Comments_Original_Submission.pdf [file giy077_response_to_reviewer_comments_original_submission.pdf]

Author's Response To Reviewer Comments

Close

Dear Editor,

Thanks to give us the opportunity to pursue the review process in GigaScience.
We answered point-by-point all the insightful reviewer comments, with a specific focus on clarification in the text, figure, and abstract. We even changed the title of the paper to make it more explicit.
We hope the revised manuscript conforms to the journal standards.

Best regards,
Yang-Min KIM, on the behalf of all the authors

**************[Reviewer comments]**************

Reviewer #1

**************Authors expressed their worry about the gaps between different version, environment, and language, which prevent bioinformatics scientists from reproducing result successfully. Moreover, they also updated their StratiPy (Python) alleviating the suffering. The proposition and ideas are good although they are hard to grasp in this paper.

It is somewhat tediously long and not informative enough.
For an example, the ABSTRACT should directly give their main result (such as the new version StratiPy) and their propositions (such as Github, Docker, Jupyter) after briefly introducing the practical need of this study. For another example, the 2.1 and 2.2 are written as a diary instead of a scientific article, there should be some tables to present the technical details and results.

If authors could rewrite the article carefully, I would like to review it again.**************

We thank the reviewer for the insightful comments and suggestions. We rewrote the manuscript, including the abstract, with the goal of clarifying the message and simplify the flow. Now the manuscript is shorter, the figure 4 has been drastically simplified and we also added a table as suggested.

We modified or added:

Title: “Experimenting with Reproducibility: a case study of Robustness in Bioinformatics”

Abstract: “Reproducibility has been shown to be limited in many scientific fields. This question is a fundamental tenet of the scientific activity, but the related issues of reusability of scientific data are poorly documented. Here, we present a case study of our difficulties to reproduce a bioinformatics method [1] although code and data were available. First, we tried to re-run the analysis with the code and data provided by the authors. Second, we

reimplemented the whole method in a Python package to avoid dependency on a MATLAB license and ease the execution of the code on HPCC (High-Performance Computing Cluster). Third, we assessed reusability of our reimplementation and the quality of our documentation, testing how easy it would be to start from our implementation to reproduce the results. In a second section, we propose solutions from this case study and other observations to improve reproducibility and research efficiency at the individual and collective level.
While finalizing our code, we created case specific documentation and tutorials for the associated Python package StratiPy. Readers are thus invited to experiment our reproducibility case study by generating the two confusion matrices of Fig 3 (see more in 2.2.2).
Here we decided to propose two options: 1) a step-by-step process to follow in a Jupyter/IPython notebook; or 2) a Docker container ready to be built and run.
Availability: last version of StratiPy (Python) with two examples of reproducibility and dataset are available at GitHub [2] and Zenodo [3]."

Page 2, line 46: [...] Robustness, “and acknowledge that new datasets or hardware environment introduce additional hurdles”

Page 2, line 50: Tackling irreproducibility [...] “, an effort that is still poorly recognized in the current publication based research community.”

Page 2, line 53: Such effort [...] “by making research products easily resuable.”

Page 4, line 99: [...] “but with different computational environment (i.e. cars), different implementation of the method (i.e. engine) and different programming languages (i.e. MATLAB and Python roads).”

Page 5, line 109: Therefore, we initially obtained very different results from the original NBS “(Fig 3 a) with heterogeneous subgroups. Once the optimal value was set up, we finally observed homogenous clusters (Fig 3 b).”

Page 6, line 140: First, our initial documentation “did not include the list of the required packages and instructions to launch the code.”

And we removed:

Page 2, line 47: “Indeed, it takes great efforts and competence to overcome all the obstacles to reproduce successfully an experiment. The process is costly in resources, both in time and funding. In computational science, there are also many technical barriers ranging from unavailable data to hardware infrastructure.”

Page 3, line 65: The authors of this study “thus should not be blamed for the difficulty that we experienced in attempting to reproduce and to make more robust their study, as they” did [...]

Page 3, line 68: “The programming code was written in MATLAB, an interpreted language originally developed for linear algebra computations which is easier and faster to write as

well as more readable than compiled language such as C, making our reproducibility attempt easier."

Page 4, line 87: it is likely [...], "and therefore should indicate how sensitive the proposed method results are with respect to these settings."

Page 5, line 110: "No or little explanation on the parameter choices can explain variability in the results as we explored the possible parameters range."

Page 5, line 120: "For instance, verifying intermediate results by plotting helped us to better understand the original code."

Page 7, line 166: "e.g. using HDF5 file instead of .mat file is more suitable to store patient's' data."

Page 7, line 179: "All these remarks are not necessarily obvious especially if the developer is working on her/his own, and to some extent "writes for her/himself"."

Page 7, line 186: "This does not in general validate the method, but at least provides a basic check."

Page 8, line 190: "Unlike theoretical and academic courses and projects, software testing systems are well developed in industry since software quality is not the priority in Academia [32]. For a student, discovering and learning this core system of reproducibility, possibly during an internship in cooperation with industry, is a great opportunity for her/his future. Furthermore, as Internet applications in science are growing, networks of scientists and developers are forming and provide learning opportunities on the development practices. For instance, software developers have recently adopted "agile" practices and fast prototyping, test based development, etc. Some of these ideas and practices can —and should— be adapted to scientific software development."

Page 8, line 205: "In a sense, we propose to use the software development test framework idea but apply it to the scientific context."

Page 8, line 213: "Scientists can use only standard data at the beginning of the project. And if there is no appropriate data, they have to suggest a new standard data."

Reviewer #2

**************The authors provide a thoughtful discussion of many of the issues related to computational reproducibility. To make their points, they work on a case study and attempt to replicate an interesting, recent, biostatistics method, network-based stratification (NBS; Hofree et al. Nature Methods 2012).
The work is interesting and helpful in clarifying many of the hidden hurdles in using code, data and reproducing results. the authors examine various aspects of software reproducibility and present challenges they faced while porting their code.
The authors also suggest best practices for both individual developers as well as for the entire community of researchers to improve reproducibility.

I have a few minor suggestions and request for clarification. But overall the work is of top quality and fit to the journal.
The authors comment on MatLab file type and HDF5. It is easy to read and write from MatLab HDF5 files. Perhaps a direct pointer to this options in that sction fo th article would allow investigators the freedom to use the programming language of choice but write data and share it using more general standards:
https://www.mathworks.com/help/matlab/ref/hdf5read.html
https://github.com/tbeu/matio
This might be important to note in the article. Especially because MatLab and Python have different types of barrier for using an algorithm for actual research. I personally see MatLab easier to teach to less code-savvy researcher. Indeed, as the authors point out Python can have its problems for sharing and reproducing because of version issue (see comment by the authors about V2 and V3). Obviously, we all wish pay-per-use licenses would go away, science would advance much faster.**************

We agree with the reviewer that Matlab is historically well designed for less code-savvy researchers and share the wish that "pay-per-use licenses would go away". The HDF5 issue was not clear and we now rewrote the part and mention:

Page 4, line 92: "For instance the data was provided by The Cancer Genome (TCGA) [23] and made available in a MATLAB .mat file format as compressed data (sparse matrices). Thanks to SciPy, Python can load all versions through v7.2 MATLAB files, but to read v7.3 .mat files, we needed an HDF5 Python library. We decided to continue using Python’s h5py package but Scipy’s sparse matrices could not be stored in HDF5 format (Table 1)."

**************A related issue is in regards to docker. The authors decided to rewrite the code in Python. An alternative would have been to dockerize the code, not re-write it. That would have allowed reproducibility and cross-platform replicability.**************

We agree that a dockerized code would allow a better reproducibility across platform but we wanted to port the method to Python to "avoid dependency on a MATLAB licence and ease the execution of the code on HPCC (High-Performance Computing Cluster)". This portability of the code was also the occasion for us to explicitly make sure to understand all the steps of the original MATLAB code. Our case study showed this was indeed required since some parts of the code were not mentioned in the method section and some parameters were arbitrarily set.

**************One recent article related to lack of reproducibility and lack of access to data can be found at.
https://www.sciencemag.org/news/2018/02/missing-data-hinder-replication-artificial-intelligence-studies It might be worth introducing the debates in reproducibility beyond in psychological and brain sciences, such issues are pervasive to science.
The work of Donoho and colleagues seems also relevant to reproducibility, for example: Buckheit, Jonathan B., and David L. Donoho. 1995. "WaveLab and Reproducible Research." In Lecture Notes in Statistics, 55-81.**************

We thank the reviewer for the suggested references. We added them in the manuscript as follow:

Page 2, line 47: "Reproducibility is a key first step, for instance, among the 400 algorithms published during the major artificial intelligence conferences, only 6% offered the code [11]."

Page 6, line 154: "In 1995, Buckheit and Donoho were already thinking about reproducible research in computer science. Their motto was “When we publish articles containing figures which were generated by computer, we also publish the complete software environment which generates the figures” by offering a complete and free package (WaveLab) to reproduce the published output [30]."

We have also learned in the book chapter of Buckheit and Donoho that Pasteur had for the first time added Methods (Material, Procedures...) section to scientific articles.

Page 9, line 229: “In the 19th century, Pasteur introduced a detailed ”Methods” section in his report: this advanced approach was necessary to reproduce his experiments and became new norms in the philosophy of science [46].”

We also added three new references:

Page 4, line 86: “it is likely that the outcomes will vary even if the same algorithm is implemented [19].”

Page 7, line 182: “In the future, journals may consider review of code as part of the standard review process [39].”

Page 9, line 222: “With considerable effort, Stodden et al. conducted a reproducibility study on 204 random articles of Science: despite some availability of the code, it had often been changed after publication, causing difficulties in replication [45]. In our proposal testing ecosystem, users will be able to launch reproduction projects more easily thanks to the code and article versioning.”

**************Figures are nice with the low-key style. Figure 4 is a bit cumbersome. I wonder whether it could be simplified or the main message clarified.**************

We simplified the Figure 4 as requested and hope it now convey the message more clearly.

Page 9, line 218: “When authors propose a new method, this method could have a reproducibility profile, which will progressively be built by authors and users (Fig 4 b.3, b.4). The information of which method does or does not work with well identified data is crucial for future work. During the optimization of a project, the software code and associated documentation should be accessible to foster collaboration on additional use cases and data. When the work achieve some level of maturity, a full fledge article can be posted on a preprint servers such as bioRxiv [43,44] and be associated with a GitHub repository by digital object identifiers (DOI). [...]
Users who test and approve reproducibility on original or new data could be credited and recognized by the scientific and developer communities (i.e. Stack Overflow, GitHub). This testing ecosystem could thus facilitate collaborations between methodology development

and biological research communities.”

New legend of Figure 4, page 15, line 425: “Figure 4: Working principles of testing ecosystem with private data. Figure 4a shows a classical case: (a.1) Authors take private data (e.g. blue data) then publish their method and corresponding results; (a.2) Users having their own data (e.g. orange data) find a relevant paper but will be lost in the labyrinth of reproducibility. Figure 4b shows testing ecosystem with standard consensus dataset: (b.1) If authors work with their own data, they must identify corresponding standard data tag(s) (e.g. blue data); (b.2) Authors initiate to develop their method with corresponding standard data and reproducibility profile will be progressively built. Bar length on iceberg corresponds to progression of replication test; (b.3) Users can test proposed method with other standard data (e.g. orange and green data) and thus participate to enhancement of the reproducibility profile; (b.4) Thanks to the collective work on testing, the method could be optimized and authors can upgrade their initial paper (versioning).”

**************Finally, that the choice of word "workflow system" accurately describes the idea introduced of standardized test dataset. Yet, "workflow" is a heavily overloaded terminology in High Performance Computing, Interface Design and other related fields. Could "regression testing" be a better term to describe what the authors are proposing?**************

We thank to reviewer for this useful comment. We renamed/replaced all the instances of “workflow system” by “testing ecosystem”. We didn’t use “regression testing” because “regression” was just one example of method for the purpose of the figure.

Close
