# Supplementary material for: Experimenting with reproducibility: a case study of robustness in bioinformatics
Source: Gigascience. 2018 Jun 28;7(7):giy077. doi: 10.1093/gigascience/giy077 (PMC6054242; doi:10.1093/gigascience/giy077)
Supplement: Response_to_Reviewer_Comments_Revision_1.pdf [file giy077_response_to_reviewer_comments_revision_1.pdf]

Author's Response To Reviewer Comments

Close

Dear Editor,

We answered point-by-point all the insightful reviewer comments, with a specific focus on clarification in the text. We hope the revised manuscript conforms to the journal standards.

Best regards,
Yang-Min KIM, on the behalf of all the authors

************** [Reviewer comments] **************

!! Conversion from word to PDF has changed some section numbers in the old version but we keep the correct numbers in this response.

Reviewer #1

************** (1)"2.2 Robustness: from MATLAB to Python" and "2.3 Reproducibility of Robustness: from Python to Python" seem have a little overlap, because in Background they were parallel concept. Please reorganize the contents in these two parts. **************

Since 2.2 is about robustness (change in code) and 2.3 is about re-running the python code on different platforms, it seems to us that this two sections can be kept separate, but the description of figure 2 has been moved from the end of paragraph 2.2.1 (Metadata and File formats, page 4, line 86) to the end of paragraph 2.2.3 (Documentation and examples, page 5, line 115) to conclude the robustness section. We also agree that the subheadings were not entirely consistent and reorganize some of the text (see below).

************** (2) The subtitles need to be more logical, for example, 2.2.1 Metadata and File formats, and 2.2.2 Codes and parameters, but 2.2.3 Jupyter/IPython. The first two subtitles are describing the effect of data, format and code, parameter on the robustness, but the "Jupyter/IPython" is not a parallel concept with the first two subtitles on the robustness, they are only platform or shell environment. Please well design the subtitles or this subsection "Jupyter/IPython" can be integrated into the 2.2.2, in brief, please make it more logical for reading. In fact, in this paper, similar problems exist several places. **************

We worked to make the logic of the text easier to follow and more consistent. In particular, we renamed several subsections like the “jupyter” subsection 2.2.3 into “Documentation and examples” (page 5, line 115), "environment" subsection 3.1.1 into "Publish software and their environment" (page 7, line 175) and "metadata" subsection 3.1.2 into "Document with appropriate Metadata" (page 7, line 187).
We also split subsection “2.3 Reproducibility of Robustness: from Python to Python” into two parts:

“2.3 Collaborative coding and best practices” (page 5, line 132) and “2.4 Reproducibility of Robustness: from Python to Python” (page 6, line 143).

************** (3) Please pay attention to the first sentence of a paragraph, it should give the main spirit of the paragraph instead of just starting a new talking. For example, "Once the environment, file format and data issues were resolved, the code was finally executed"… For another example, "Given the observed difficulties, in this section we draw some conclusions on this reproducibility case study experiment and suggest some tools and best practices.", why always "some conclusion"? why cannot directly summarize the conclusion here? Another example, "3.1.1 Environment In 1995, Buckheit and Donoho were already thinking about reproducible research in computer science", this is a composition or fiction genre instead of a scientific paper.
(4) After rewriting all first sentences for each paragraphs, please reorganize the content of their following sentences referring to other published scientific articles. **************

We rewrote the first sentences of the paragraph across the paper.

Page 4, line 100, paragraph 2.2.2 Codes and parameters: we changed the sentence "Once the environment, file format and data issues were resolved, the code was finally executed" into "Beyond documentation and file formats, code initialization and parameters settings are also key for reproducibility."

Page 5, line 133, paragraph 2.3 Collaborative code development and best practices: “Throughout the project we used the version control system (VCS) Git to document the development of our Python package.”

Page 6, line 144, paragraph 2.4 Reproducibility of Robustness: from Python to Python: “Knowing how difficult it can be to re-run someone else’s code, we then attempted to start the analysis from scratch and to reproduce the results on another platform from our newly developed python package.”

Page 7, line 172, paragraph 3.1 Act locally: simple practices and available tools: we replaced the sentence "Given the observed difficulties, in this section we draw some conclusions on this reproducibility case study experiment and suggest some tools and best practices." by “We conclude from this reproducibility case study experiment by suggesting tools and best practices following the programming best practices”.

Page 7, line 176, paragraph 3.1.1 Publish software and their environment: regarding the sentence about Buckheit and Donoho, we totally rewrote it as follow: "Increased reproducibility and replicability can be obtained by following Buckheit and Donoho’s long standing motto: “When we publish articles containing figures which were generated by computer, we also publish the complete software environment which generates the figures” by offering a complete and free package (WaveLab) to reproduce the published output [30]."

Page 7, line 195, paragraph 3.1.3 Write readable code: we changed the sentence “Anyone who has spent time to understand someone else’s code would advise some simple basic rules to help make the code readable and understandable.” into “We draw some conclusion from our experience in working with others code.”. We then follow the reviewer advice to directly summarize the conclusion.

************** (5) Based on the size of core content of this article, please cut it down. **************

We removed some of the text to make it more dense.. Especially in the conclusion, we adopt a straightforward bullet-point list of key messages and recommandations:

Page 11, line 297: "To summarize, our experiment at reproducing initial results led to the following conclusions and recommendations:
- Improve life scientists software development skills
- Use online repositories and tools to help other scientists in their exploration of the method [26,27,31]
- Enhance the cooperation between academic education and industry [40,41,47]
- Develop an open source continuous testing ecosystem with community standards, well-identified datasets to validate tools across versions and datasets, and go beyond the publication of a PDF file”

In total we have reduced 217 words.

Reviewer #2

************** The authors have successfully responded to my comments. I congratulate the authors for a simple and nice paper. **************

We thank reviewer 2 for all his helpful comments and interest in our paper.

Close
