# Supplementary material for: Experimenting with reproducibility: a case study of robustness in bioinformatics
Source: Gigascience. 2018 Jun 28;7(7):giy077. doi: 10.1093/gigascience/giy077 (PMC6054242; doi:10.1093/gigascience/giy077)
Supplement: Reviewer_1_Report_(Revision_1) -- Yi Zhang — 4/28/2018 Reviewed [file giy077_reviewer_1_report_(revision_1).pdf]

Reviewer Report

Title: **Experimenting with Reproducibility: a case study of Robustness in Bioinformatics**

Version: **Revision 1** **Date** 28-April-2018

Reviewer name: **Yi Zhang**

Reviewer Comments to Author: I see the improvement in abstract and Table 1, but I still feel it is a little tedious,
(1)"2.2 Robustness: from MATLAB to Python" and "2.3 Reproducibility of Robustness: from Python to Python" seem have a little overlap, because in Background they were parallel concept. Please reorganize the contents in these two parts.
(2) The subtitles need to be more logical, for example, 2.2.1 Metadata and File formats, and 2.2.2 Codes and parameters, but 2.2.3 Jupyter/IPython. The first two subtitles are describing the effect of data, format and code, parameter on the robustness, but the "Jupyter/IPython" is not a parallel concept with the first two subtitles on the robustness, they are only platform or shell environment. Please well design the subtitles or this subsection "Jupyter/IPython" can be integrated into the 2.2.2, in brief, please make it more logical for reading. In fact, in this paper, similar problems exist several places.
(3)Please pay attention to the first sentence of a paragraph, it should give the main spirit of the paragraph instead of just starting a new talking. For example, "Once the environment, file format and data issues were resolved, the code was finally executed"… For another example, "Given the observed difficulties, in this section we draw some conclusions on this reproducibility case study experiment and suggest some tools and best practices.", why always "some conclusion"? why cannot directly summarize the conclusion here? Another example, "3.1.1 Environment In 1995, Buckheit and Donoho were already thinking about reproducible research in computer science", this is a composition or fiction genre instead of a scientific paper.
(4)After rewriting all first sentences for each paragraphs, please reorganize the content of their following sentences referring to other published scientific articles.
(5)Based on the size of core content of this article, please cut it down.

Level of Interest

Please indicate how interesting you found the manuscript: Choose an item.

Quality of Written English

Please indicate the quality of language in the manuscript: Choose an item.

Declaration of Competing Interests

Please complete a declaration of competing interests, considering the following questions:

- Have you in the past five years received reimbursements, fees, funding, or salary from an organisation that may in any way gain or lose financially from the publication of this manuscript, either now or in the future?
- Do you hold any stocks or shares in an organisation that may in any way gain or lose financially from the publication of this manuscript, either now or in the future?
- Do you hold or are you currently applying for any patents relating to the content of the manuscript?
- Have you received reimbursements, fees, funding, or salary from an organization that holds or has applied for patents relating to the content of the manuscript?
- Do you have any other financial competing interests?
- Do you have any non-financial competing interests in relation to this paper?

If you can answer no to all of the above, write 'I declare that I have no competing interests' below. If your reply is yes to any, please give details below.

I declare that I have no competing interests

I agree to the open peer review policy of the journal. I understand that my name will be included on my report to the authors and, if the manuscript is accepted for publication, my named report including any attachments I upload will be posted on the website along with the authors' responses. I agree for my report to be made available under an Open Access Creative Commons CC-BY license (http://creativecommons.org/licenses/by/4.0/). I understand that any comments which I do not wish to be included in my named report can be included as confidential comments to the editors, which will not be published.

I agree to the open peer review policy of the journal

To further support our reviewers, we have joined with Publons, where you can gain additional credit to further highlight your hard work (see: https://publons.com/journal/530/gigascience). On publication of this paper, your review will be automatically added to Publons, you can then choose whether or not to claim your Publons credit. I understand this statement. Yes
