# Supplementary material for: Experimenting with reproducibility: a case study of robustness in bioinformatics
Source: Gigascience. 2018 Jun 28;7(7):giy077. doi: 10.1093/gigascience/giy077 (PMC6054242; doi:10.1093/gigascience/giy077)
Supplement: Reviewer_1_Report_(Original_Submission) -- Yi Zhang — 1/31/2018 Reviewed [file giy077_reviewer_1_report_(original_submission).pdf]

Reviewer Report

Title: **Experimenting with Reproducibility: a case study of Robustness in Bioinformatics**

Version: **Original Submission** **Date:** 1/31/2018

Reviewer name: **Yi Zhang**

Reviewer Comments to Author:

Authors expressed their worry about the gaps between different version, environment, and language, which prevent bioinformatics scientists from reproducing result successfully. Moreover, they also updated their StratiPy (Python) alleviating the suffering. The proposition and ideas are good although they are hard to grasp in this paper. It is somewhat tediously long and not informative enough. For an example, the ABSTRACT should directly give their main result (such as the new version StratiPy) and their propositions (such as Github, Doker, Jupyter) after briefly introducing the practical need of this study. For another example, the 2.1 and 2.2 are written as a diary instead of a scientific article, there should be some tables to present the technical details and results. If authors could rewrite the article carefully, I would like to review it again.

Level of Interest

Please indicate how interesting you found the manuscript: An article of limited interest

Quality of Written English

Please indicate the quality of language in the manuscript: Needs some language corrections before being published

Declaration of Competing Interests

Please complete a declaration of competing interests, considering the following questions:

- Have you in the past five years received reimbursements, fees, funding, or salary from an organisation that may in any way gain or lose financially from the publication of this manuscript, either now or in the future?
- Do you hold any stocks or shares in an organisation that may in any way gain or lose financially from the publication of this manuscript, either now or in the future?
- Do you hold or are you currently applying for any patents relating to the content of the manuscript?
- Have you received reimbursements, fees, funding, or salary from an organization that holds or has applied for patents relating to the content of the manuscript?

- Do you have any other financial competing interests?
- Do you have any non-financial competing interests in relation to this paper?

If you can answer no to all of the above, write 'I declare that I have no competing interests' below. If your reply is yes to any, please give details below.

I declare that I have no competing interests

I agree to the open peer review policy of the journal. I understand that my name will be included on my report to the authors and, if the manuscript is accepted for publication, my named report including any attachments I upload will be posted on the website along with the authors' responses. I agree for my report to be made available under an Open Access Creative Commons CC-BY license (http://creativecommons.org/licenses/by/4.0/). I understand that any comments which I do not wish to be included in my named report can be included as confidential comments to the editors, which will not be published.

I agree to the open peer review policy of the journal

To further support our reviewers, we have joined with Publons, where you can gain additional credit to further highlight your hard work (see: https://publons.com/journal/530/gigascience). On publication of this paper, your review will be automatically added to Publons, you can then choose whether or not to claim your Publons credit. I understand this statement. Yes
