# Supplementary material for: Experimenting with reproducibility: a case study of robustness in bioinformatics
Source: Gigascience. 2018 Jun 28;7(7):giy077. doi: 10.1093/gigascience/giy077 (PMC6054242; doi:10.1093/gigascience/giy077)
Supplement: Reviewer_2_Report_(Original_Submission) -- Franco Pestilli — 2/25/2018 Reviewed [file giy077_reviewer_2_report_(original_submission).pdf]

Reviewer Report

Title: **Experimenting with Reproducibility: a case study of Robustness in Bioinformatics**

Version: **Original Submission** **Date:** 2/25/2018

Reviewer name: **Franco Pestilli**

Reviewer Comments to Author:

The authors provide a thoughtful discussion of many of the issues related to computational reproducibility. To make their points, they work on a case study and attempt to replicate an interesting, recent, biostatistics method, network-based stratification (NBS; Hofree et al. Natur Methods 2012).

The work is interesting and helpful in clarifying many of the hidden hurdles in using code, data and reproducing results. the authors examine various aspects of software reproducibility and present challenges they faced while porting their code.

The authors also suggest best practices for both individual developers as well as for the entire community of researchers to improve reproducibility.

I have a few minor suggestions and request for clarification. But overall the work is of top quality and fit to the journal.

The authors comment on MatLab file type and HDF5. It is easy to read and write from MatLab HDF5 files. Perhaps a direct pointer to this options in that sction fo th article would allow invstigators the freeedom to use the programming language of choice but write data and share it using mor genral standards:
https://www.mathworks.com/help/matlab/ref/hdf5read.html
https://github.com/tbeu/matio

This might be important to note in the article. Especially because MatLab and Python have different types of barrier for using an algorithm for actual research. I personally see MatLab easier to teach to less code-savvy researcher. Indeed, as the authors point out Python can have its problems for sharing and reproducing because of version issue (see comment by the authors about V2 and V3). Obviously, we all wish pay-per-use licenses would go away, science would advance much faster.

A related issue is in regards to docker. The authors decided to rewrite the code in Python. An alternative would have been to dockerize the code, not re-write it. That would have allowed reproducibility and cross-platform replicability.

One recent article related to lack of reproducibility and lack of access to data can be found at. https://www.sciencemag.org/news/2018/02/missing-data-hinder-replication-artificial-intelligence-studies It might be worth introducing the debates in reproducibility beyond in psychological and brain sciences, such issues are pervasive to science.

The work of Donoho and colleagues seems also relevant to reproducibility, for example: Buckheit, Jonathan B., and David L. Donoho. 1995. "WaveLab and Reproducible Research." In Lecture Notes in Statistics, 55-81.

Figures are nice with the low-key style. Figure 4 is a bit cumbersome. I wonder whether it could be simplified or the main message clarified.

Finally, that the choice of word "workflow system" accurately describes the idea introduced of standardized test dataset. Yet, "workflow" is a heavily overloaded terminology in High Performance Computing, Interface Design and other related fields. Could "regression testing" be a better term to describe what the authors are proposing?

Level of Interest

Please indicate how interesting you found the manuscript: An article of importance in its field

Quality of Written English

Please indicate the quality of language in the manuscript: Acceptable

Declaration of Competing Interests

Please complete a declaration of competing interests, considering the following questions:

- Have you in the past five years received reimbursements, fees, funding, or salary from an organisation that may in any way gain or lose financially from the publication of this manuscript, either now or in the future?
- Do you hold any stocks or shares in an organisation that may in any way gain or lose financially from the publication of this manuscript, either now or in the future?
- Do you hold or are you currently applying for any patents relating to the content of the manuscript?
- Have you received reimbursements, fees, funding, or salary from an organization that holds or has applied for patents relating to the content of the manuscript?
- Do you have any other financial competing interests?
- Do you have any non-financial competing interests in relation to this paper?

If you can answer no to all of the above, write 'I declare that I have no competing interests' below. If your reply is yes to any, please give details below.

I declare that I have no competing interests

I agree to the open peer review policy of the journal. I understand that my name will be included on my report to the authors and, if the manuscript is accepted for publication, my named report including any attachments I upload will be posted on the website along with the authors' responses. I agree for my report to be made available under an Open Access Creative Commons CC-BY license (http://creativecommons.org/licenses/by/4.0/). I understand that any comments which I do not wish to be included in my named report can be included as confidential comments to the editors, which will not be published.

I agree to the open peer review policy of the journal

To further support our reviewers, we have joined with Publons, where you can gain additional credit to further highlight your hard work (see: https://publons.com/journal/530/gigascience). On publication of this paper, your review will be automatically added to Publons, you can then choose whether or not to claim your Publons credit. I understand this statement. Yes
